# Mosses and Snails as Bioindicators Reflecting the Biologically Relevant Fraction of Toxic Elements

**DOI:** 10.3390/ijms27125225

**Published:** 2026-06-09

**Authors:** Alessia Postiglione, Alessia Di Fraia, Tania Russo, Gianluca Polese, Martina Dentato, Sergio Sorbo, Adriana Basile, Viviana Maresca

**Affiliations:** 1 Department of Biology, University of Naples Federico II, 80126 Naples, Italy; alessia.postiglione@unina.it (A.P.); alessia.difraia@unina.it (A.D.F.); gianluca.polese@unina.it (G.P.); martina.dentato@unina.it (M.D.); adbasile@unina.it (A.B.); 2 Department of Biology, Ecology and Earth Science, University of Calabria, 87036 Rende, Italy; 3 Centro Servizi Metrologici e Tecnologici Avanzati (CeSMA), Section of Microscopy, University of Naples Federico II, 80126 Naples, Italy; sersorbo@unina.it; 4 Department of Life Science, Health, and Health Professions, Link Campus University, 00165 Rome, Italy; v.maresca@unilink.it

**Keywords:** air pollution, toxic elements, bioindicators, oxidative stress biomarkers, histopathology

## Abstract

Air pollution is a major environmental and public health issue, largely driven by human activities. The present study evaluates the combined use of two bioindicators from different taxonomic groups, the moss *Rhytidiadelphus squarrosus* and the terrestrial snail *Cornu aspersum*, to assess early biological effects induced by atmospheric exposure to toxic elements. Both species, chosen for their sensitivity, simple physiology, and suitability for field transplantation, were exposed for 30 days at two sites in southern Italy with contrasting environmental conditions. Toxic element accumulation in moss biomass and snail tissues was measured using ICP-OES, while snail shell composition was analyzed using FTIR spectroscopy. Biological responses were assessed through oxidative stress biomarkers (ROS levels and catalase activity), HSP70 expression determined by Western blotting, and structural damage, including ultrastructural changes in mosses and histopathological alterations in snails. Results showed site-dependent patterns of toxic elements accumulation in both organisms, consistent with increased oxidative stress and induction of HSP70 expression. Enlargement of the albumen gland and histological alterations in digestive tubules and reproductive systems were found in snails. Mosses showed severe ultrastructural alterations. FTIR analysis revealed changes in snail shell composition consistent with metal exposure. Principal component analysis highlighted clear patterns linking contamination, oxidative stress, and structural damage, supporting the complementarity of the two bioindicators and their ability to capture distinct exposure pathways and biological effects.

## 1. Introduction

Air pollution poses a significant threat to the environment and public health due to its chronic impact on ecosystems and human populations, particularly through chronic exposure to toxic elements [1,2]. Elements such as arsenic, a metalloid, and heavy metals such as cadmium, nickel, and lead are of particular concern due to their persistence, non-biodegradability, and capacity for bioaccumulation and biomagnification, resulting in long-term risks for both the environment and human health [3,4]. Chronic exposure to these substances has been linked to carcinogenic, neurotoxic, reproductive, and developmental effects, especially in vulnerable populations [5,6]. Despite the existence of regulatory frameworks such as WHO guidelines and Legislative Decree 155/2010 in Italy, continuous emissions from industrial activities, traffic, agriculture, and improper waste disposal maintain these elements in the atmosphere [7]. While conventional air monitoring systems provide accurate concentration measurements, they have limitations in terms of spatial resolution and do not consider the bioavailability of contaminants, their combined effects, or early biological responses at the organism level [3,8]. For this reason, complementary biological approaches may be useful for capturing the ecological relevance of atmospheric contamination.

Studies based on bioindicators integrate multiple exposure pathways and reflect biologically relevant contamination [9,10]. However, most studies rely on single taxonomic groups, focusing on atmospheric deposition or internal bioaccumulation and toxicity, rarely linking contamination to effects at the organism level across different taxa. This compartmentalized approach may represent a limitation in understanding the complex nature of contamination impacts and in developing sensitive assessment strategies. However, several studies have demonstrated the effectiveness of individual bioindicator organisms in detecting toxic element contamination and its effects. The study conducted by [11] demonstrated that the moss *Scorpiurum circinatum* can detect airborne toxic elements following waste burning through bioaccumulation processes and the activation of antioxidant enzymes. Similarly, De Vaufleury and Pihan [12] demonstrated that snails accumulate metals such as cadmium, lead, copper, and zinc in their tissues. Thus, snails provide information on the environmental bioavailability of contaminants and activate detoxifying enzymes such as glutathione S-transferase (GST) [13]. Nevertheless, studies simultaneously integrating the responses of both plant and animal organisms exposed to the same environmental conditions remain limited. A joint understanding of these responses could provide a more comprehensive assessment of the effects of toxic elements on the ecosystem.

To address this issue, the present study applies an integrated plant–animal approach, combining the use of the moss *Rhytidiadelphus squarrosus* and the snail *Cornu aspersum*. Mosses effectively accumulate airborne toxic elements due to their morphology and exclusive dependence on atmospheric deposition, and the moss bag technique allows for standardized spatial comparisons. Their rapid physiological responses to environmental stress also make them suited to detecting early changes in air quality [14,15,16,17,18]. Snails, meanwhile, provide complementary information on internal bioavailability, specific bioaccumulation in target tissues, and toxicological consequences, thereby linking environmental contamination to potentially biological impairment at higher trophic levels [12,13]. The study was conducted in southern Italy at two sites characterized by different levels of anthropogenic pressure. The ‘Land of Fires’ area, including Giugliano in Campania (GC), was selected as an area characterized by chronic atmospheric contamination linked to illegal waste disposal and burning, with documented consequences for the environment and human health [19]. In contrast, Montemiletto (MM) is a rural, inland area with low levels of urbanization and no major sources of pollution; it was chosen as a site with reduced environmental pressure. These real-world scenarios provide an appropriate framework for assessing the sensitivity of a bi-taxonomic assessment approach under contrasting exposure conditions. This experimental design provides insight into the value of simultaneously studying plants and animals under the same field conditions. The objectives of the study are: (*i*) to evaluate the bioaccumulation of toxic elements in mosses and snails in contrasting environmental contexts while exploring the feasibility of an integrated plant–animal approach; (*ii*) to assess oxidative stress responses (ROS and catalase) in both organisms, together with HSP70 expression and histopathological alterations in the hepatopancreas and ovotestis of snails and ultrastructural changes in moss cells; and (*iii*) to evaluate FTIR spectral variations in snail shells as potential indicators of environmental toxic elements exposure.

## 2. Results

### 2.1. Bioaccumulation

**Figure 1:**
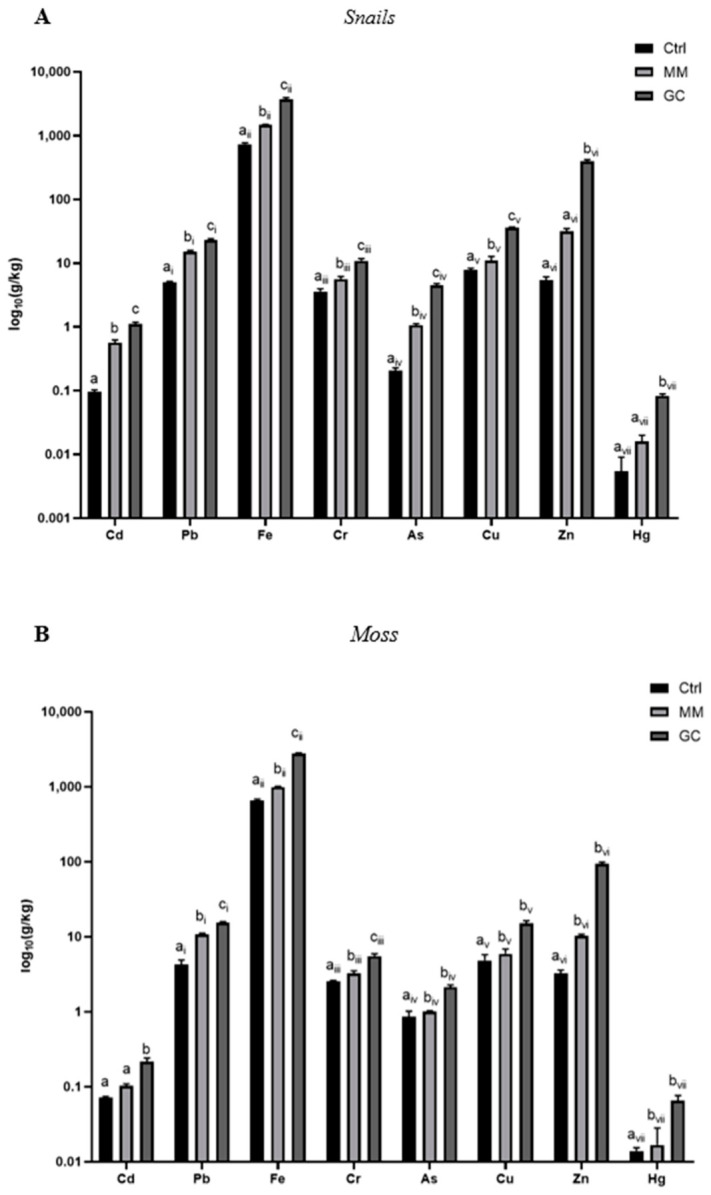
Heavy metal concentrations (log_10_ g/kg) detected in *Cornu aspersum* (**A**) and *Rhytidiadelphus squarrosus* (**B**) at the control site (Ctrl) and after 30 days at the MM and GC sites. Values are expressed as cage-level means ± SD (*n* = 3 cages per site). For each element, different letters indicate significant differences among sites (*p* < 0.05, Tukey’s HSD test).

Higher levels (log_10_ g/kg) of iron (Fe), zinc (Zn), copper (Cu), cadmium (Cd), lead (Pb), chromium (Cr), arsenic (As), and mercury (Hg) were detected at the exposed sites compared with the control site, with the highest concentrations found in GC samples (Figure 1). These findings suggest higher levels of environmental contamination in the GC area than in MM and the control site. Element bioaccumulation (g/kg) is reported in Figure S1.

Pearson correlation heatmaps revealed distinct association patterns between moss and snail samples depending on the analyzed element, with particularly high positive correlations for Zn and Cr and strong negative correlations for Cd, Pb, and Cu under specific treatment combinations. The Pearson correlation values between the bioaccumulation of each element in *R. squarrosus* and *C. aspersum* are reported in Figure S2 and refer to cage-level means (*n* = 3 cages per site).

Although metal accumulation was generally higher in snails than in mosses, the relative distribution of toxic elements among the three sites (control, MM and GC) showed similar accumulation proportions and patterns in both organisms (see Figure 1).

### 2.2. FTIR Analysis

**Figure 2:**
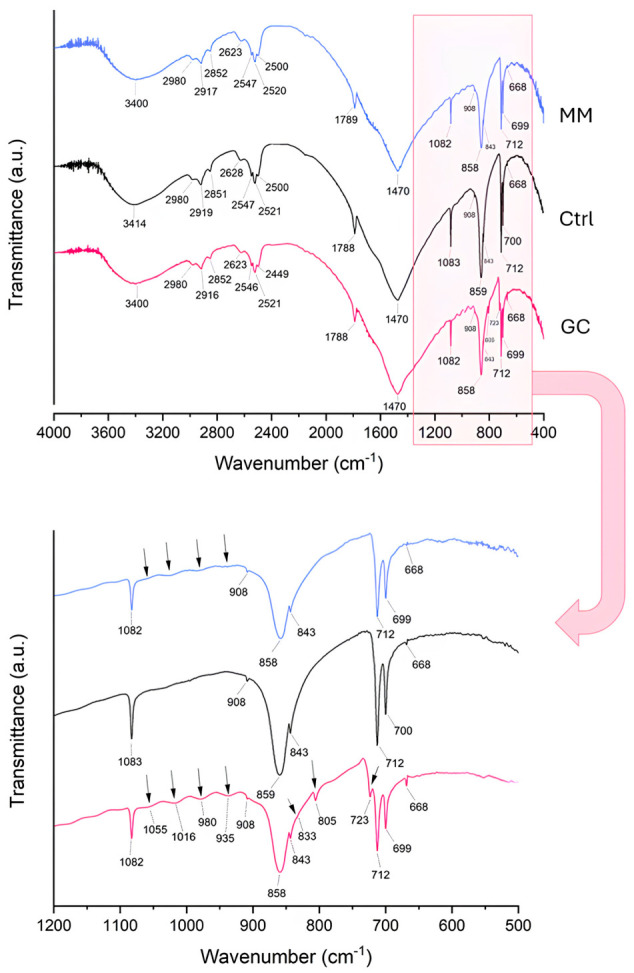
FT-IR spectra of snail shells from MM (blue), Ctrl (black), and GC (pink). Black arrows indicate additional peaks not detected in the Ctrl samples.

Figure 2 shows the results of the FTIR analysis of snail shells, which provided indirect evidence of structural changes potentially associated with toxic element exposure. All spectra showed a main absorption band at 1470 cm^−1^, associated with carbonate ions (CO_3_^2−^). Secondary peaks were also detected at 712, 858, 1082, and 1789 cm^−1^, which are characteristic of the aragonite structure. A band between 3400 and 3600 cm^−1^, attributable to the stretching of hydroxyl (OH) groups, was also detected and was presumably associated with organic residues in the powdered samples. Significant differences emerged when the spectra of the three groups (CTRL, MM, and GC) were compared in the fingerprint region (935–1055 cm^−1^): four clear peaks appeared in the GC and MM samples that were absent in the control. Three additional peaks (at 833, 805, and 723 cm^−1^) appeared in the GC samples only. Significant reductions in signal intensity were observed at 1470, 858, 712, and 699 cm^−1^ in the contaminated samples compared with the control.

### 2.3. Biological Responses: ROS Levels and CAT Activity

#### 2.3.1. Reactive Oxygen Species

**Figure 3:**
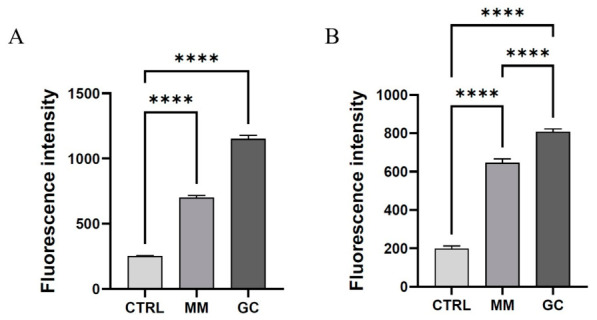
ROS production expressed as fluorescence intensity of DCFDH measured in the snail *Cornu aspersum* (**A**) and the moss *Rhytidiadelphus squarrosus* (**B**) at the control sites, MM, and GC, after 30 days of exposure. Asterisks mark the level of significance (One-Way ANOVA) (**** *p* < 0.0001). Statistical analyses were performed using cage-level means (*n* = 3 cages per site).

The production of ROS was quantified fluorometrically in *C. aspersum* and *R. squarrosus*. In *C. aspersum* (Figure 3A), the fluorescence intensity increased significantly in both the MM and the GC groups compared with the control group. Furthermore, the levels of ROS in the GC group were higher than in the MM group in snails, although the differences were not statistically significant (Figure 3A). A similar trend was observed in *R. squarrosus* (Figure 3B), where both treated groups showed a significant increase in fluorescence intensity compared with the control. The GC group showed significantly higher values than the MM group.

#### 2.3.2. Catalase Activity

**Figure 4:**
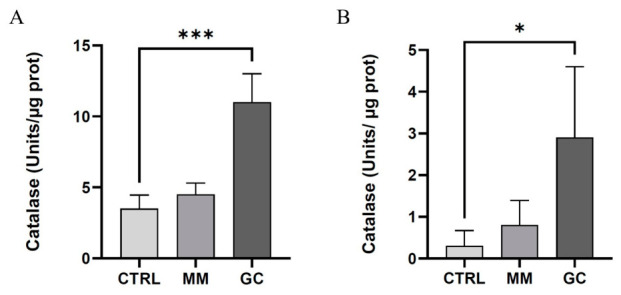
The histogram shows the mean ± SD values (units/μg prot) of CAT enzyme activity in the snail *Cornu aspersum* (**A**) and the moss *Rhytidiadelphus squarrosus* (**B**) at the control sites, MM, and GC after 30 days of exposure. Asterisks mark the level of significance (One-Way ANOVA) (* *p* < 0.05, *** *p* < 0.001). Statistical analyses were performed using cage-level means (*n* = 3 cages per site).

The CAT activity was measured in *C. aspersum* and *R. squarrosus* samples from three different sites to assess the oxidative stress induced by heavy metal contamination. In snail samples (Figure 4A), the lowest catalase activity values were measured in the control samples (3.58 units/μg protein), while significantly higher values were found in samples taken from MM (4.23 units/μg protein) and GC (11.02 units/μg protein) compared with the control samples. Similar differences were also observed in moss samples, where CAT activity showed significant variability between sites (Figure 4B). The lowest values were recorded at the control site, with a median value of 0.413 units/μg of protein. Higher values were found in samples from MM (0.916 units/μg protein) and GC (3.090 units/μg protein), the latter of which was significantly higher than the control.

### 2.4. HSP70 Expression in R. squarrosus and C. aspersum

**Figure 5:**
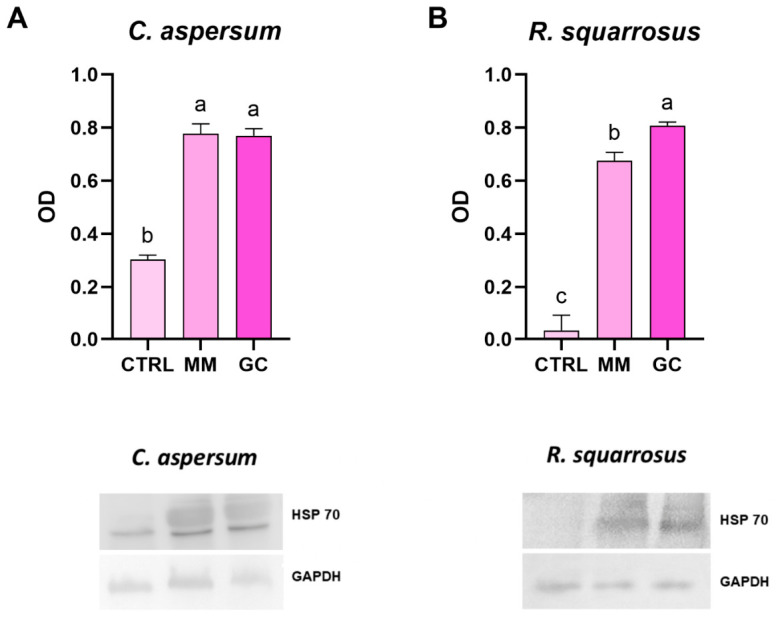
Expression of HSP70 protein in *C. aspersum* (**A**) and *R. squarrosus* (**B**) under the different experimental conditions. Graphs show optical density (OD) values corresponding to HSP70 expression in CTRL, MM, and GC groups. HSP70 concentration is shown relative to the GAPDH level. Bars represent cage-level means ± SD (*n* = 3 cages per site). Different letters indicate statistically significant differences among groups (*p* < 0.05). Representative Western blot images for HSP70 and GAPDH are shown below each graph.

Heat Shock Protein 70 (HSP70) expression showed species-specific responses to the experimental treatments. In *C. aspersum* (Figure 5A), both MM and GC treatments induced a marked increase in HSP70 levels compared with the CTRL group (*p* < 0.05). The two treated groups exhibited comparable expression levels, indicating a similar induction of the stress response pathway. Densitometric analysis revealed approximately a 2.5-fold increase in HSP70 expression in MM and GC specimens relative to controls.

In *R. squarrosus* (Figure 5B), a progressive increase in HSP70 expression was observed across the experimental groups. The CTRL group showed very low basal levels of HSP70, whereas MM treatment significantly enhanced protein expression (*p* < 0.05). The highest HSP70 levels were detected in the GC group, which differed significantly from both CTRL and MM groups (*p* < 0.05), suggesting a stronger activation of cellular stress-response mechanisms under GC exposure.

The Western blot profiles were consistent with the densitometric data, showing stronger HSP70 immunoreactive bands in treated specimens, while GAPDH expression remained stable among groups, confirming equal protein loading and reliable normalization.

### 2.5. Snails’ Histological Changes and Histopathological Index (I_h_) and Albumen Gland Evaluation

#### 2.5.1. Digestive Glands

**Figure 6:**
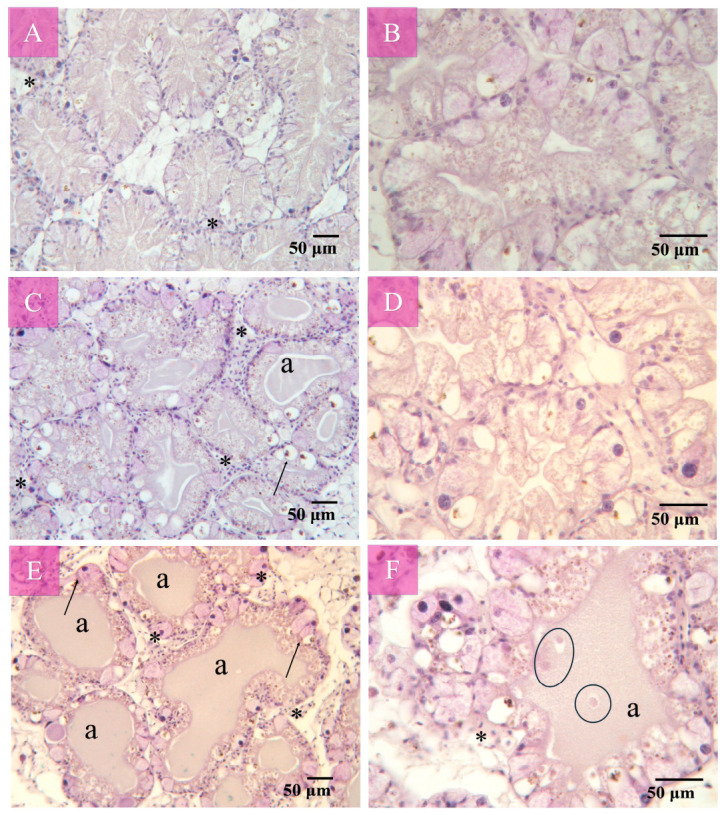
Representative light micrographs of *Cornu aspersum* digestive tubules (**A**–**F**) in bright field. Panels (**A**,**C**,**E**): 20×; panels (**B**,**D**,**F**): 40×. In control samples (**A**,**B**), tubules show regular lumens and the presence of intertubular hemocytes (*). Digestive tubules after 30 days of exposure at MM (**C**,**D**) show an increase in excretory cells (arrows), with the presence of tubules with regular lumens and some atrophy (a) of the tubular lumen. Digestive tubules after 30 days of exposure at GC (**E**,**F**) show abundant hemocyte infiltration (*) in the intertubular space, the presence of cellular debris (circles), and an increase in excretory cells (arrows). Scale bars: 50 µm.

Histological analysis revealed that the control samples exhibited a healthy and intact digestive gland structure. The only detectable alteration was a slight intertubular hemocyte infiltration, which is considered physiological (Figure 6A,B). After 30 days of exposure, slight atrophy of the tubular lumen and an increase in excretory cells were observed in samples from MM. Moderate infiltration of intertubular hemocytes was also present in this case (Figure 6C,D). Samples exposed to GC for 30 days showed the most compromised histological picture (Figure 6E,F). These alterations included increased infiltration of intertubular hemocytes, tubule atrophy with irregularly shaped surrounding cells and increased intertubular space and cellular debris. No cellular necrosis was observed in any sample, including those from GC.

#### 2.5.2. Ovotestis

**Figure 7:**
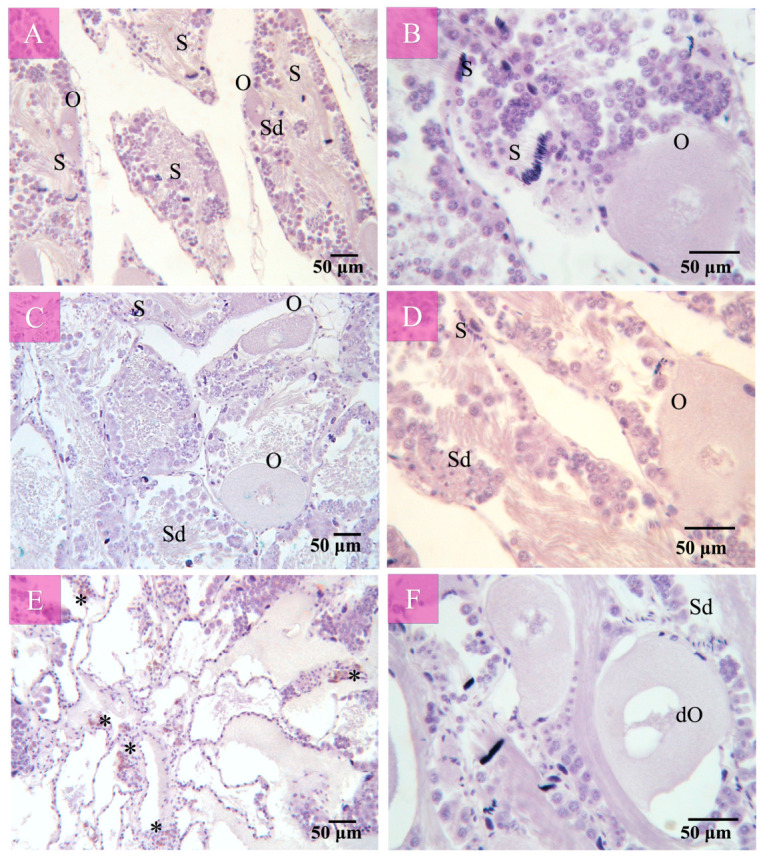
Representative light micrographs of *Cornu aspersum* ovotestis (**A**–**F**) in bright field. Panels (**A**,**C**,**E**): 20×; panels (**B**,**D**,**F**): 40×. Panels (**A**,**B**) show control follicles with gametes at different stages of maturation. Panels (**C**,**D**) show MM follicles containing abundant gametes. Panel (**E**) shows GC snails’ empty follicles with lipofuscin aggregates (*). Panel (**F**) shows GC gonads with deformed oocytes (dO). S, spermatozoa; Sd, spermatids; O, oocytes. Scale bars: 50 µm.

Histological examination of the ovotestis in the control group revealed numerous follicles containing male and female gametes at various stages of maturation (Figure 7A,B). A comparable follicular structure with preserved gametogenic progression was observed in snails exposed to MM for 30 days (Figure 7C,D). By contrast, snails exposed to GC for the same period exhibited significant gonadal alterations (Figure 7E,F). The follicles appeared mostly empty and contained lipofuscin aggregates. Reduced gametogenic activity was evident, with acini displaying degenerated gametes and disorganized architecture.

#### 2.5.3. Histopathological Index

The histopathological index (*Iₕ*) showed a clear increase in the two analyzed organs (the digestive tubules and the ovotestis) in relation to the toxic element contamination levels in the studied sites (Table 1). In the digestive tubules, the average *I_h_* values were 0.20 ± 0.04 at the control site (CTRL), 0.36 ± 0.07 at the moderately contaminated site (MM), and 0.77 ± 0.11 at the severely contaminated site (GC). This corresponds to a classification of histological damage ranging from low to very high, respectively. A similar trend was observed in the ovotestis, with average *I_h_* values of 0.10 ± 0.02 (CTRL), 0.29 ± 0.02 (MM), and 0.73 ± 0.04 (GC).

#### 2.5.4. Albumen Gland Length Evaluation

**Figure 8:**
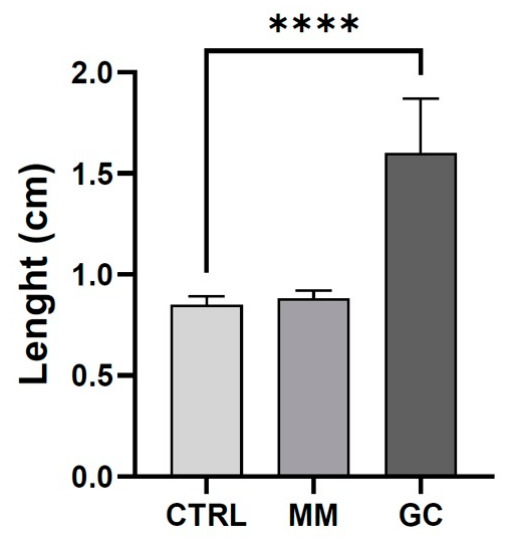
Histogram showing the mean ± SD values of albumen gland length in *Cornu aspersum* snails. Asterisks mark the level of significance (One-Way ANOVA) (**** *p* < 0.0001).

A significant increase in albumen gland size was observed in snails from the GC site compared with MM and control groups. The albumen gland size showed mean values of 0.85 ± 0.04 cm in CTRL, 0.88 ± 0.04 cm in MM, and 1.60 ± 0.27 cm in GC (Figure 8).

### 2.6. TEM: Moss Ultrastructural Damage Index (UDI)

**Figure 9:**
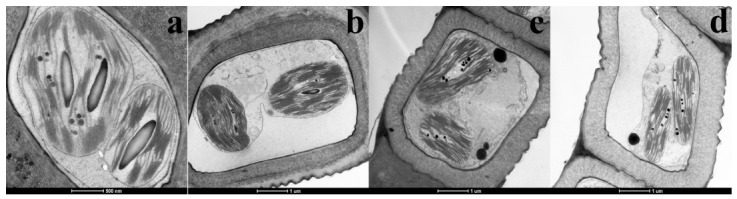
The figure shows micrographs from leaflet cells of moss samples collected from Monte Nuovo (**a**) and exposed at MM (**b**) and GC (**c**,**d**). (**a**) Sample from Monte Nuovo. A leaflet cell, delimited by a thick cell wall, contains two chloroplasts, provided with well-developed thylakoid systems with grana and intergrana thylakoids. Starch grains and a few plastoglobules are also visible in the stroma of the chloroplasts. (**b**) Sample exposed at MM. The micrograph shows a highly plasmolysed cell with two chloroplasts featuring a control-like appearance. (**c**,**d**) show samples exposed at GC. (**c**) shows a protoplast with two well-developed chloroplasts and large electron-dense droplets in the cytoplasm. (**d**) shows a severely plasmolyzed cell with two well-developed chloroplasts and a large electron-dense droplet in the cytoplasm. Scale bars are reported below the micrographs.

The samples from Monte Nuovo (CTRL) exhibited characteristic ultrastructural features. The chloroplasts were oblong and contained well-developed thylakoid systems, with grana and intergrana thylakoids that extending along the organelle’s main axis. A few plastoglobules and starch grains were present in the stroma of the chloroplasts (Figure 9a). Samples exposed at MM showed plasmolysis of the cells (Figure 9b). Samples exposed at GC exhibited both plasmolysis and the presence of electron-dense droplets in the cytoplasm (Figure 9c,d). As observed with *I_h_*, the Ultrastructural Damage Index (UDI) calculated for mosses showed a clear progression of cellular damage with increasing contamination. In the chloroplast compartment, mean UDI values were 0.15 ± 0.03 (CTRL), 0.38 ± 0.05 (MM), and 0.82 ± 0.06 (GC), corresponding to a damage classification ranging from low to very high, respectively. In the cytoplasmic/membrane compartment, the values were 0.18 ± 0.02 (CTRL), 0.41 ± 0.04 (MM), and 0.85 ± 0.05 (GC), confirming a significant increase in the spread of ultrastructural alterations in contaminated sites (Table 2).

### 2.7. Principal Component Analysis (PCA)

**Figure 10:**
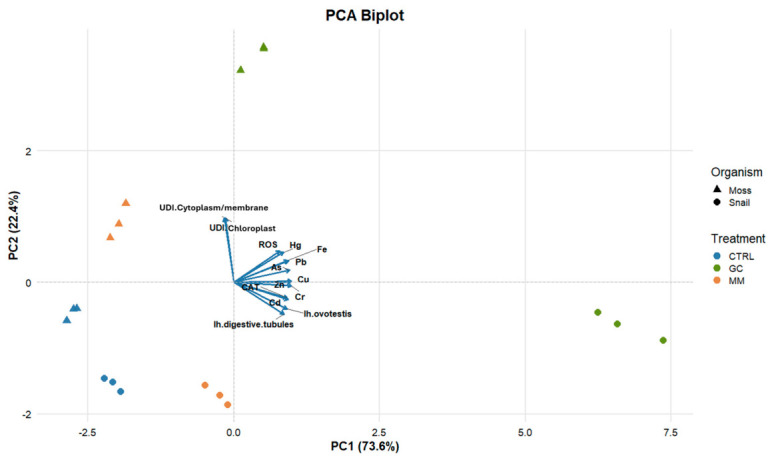
PCA biplot showing the distribution of moss (triangles) and snail (circles) samples under different treatments: control (CTRL, blue), GC (green), and MM (orange). Principal Component 1 (PC1) explains 73.6% of the total variance, while Principal Component 2 (PC2) explains 22.4%. Vectors represent the contribution and direction of measured variables, including heavy metals (Fe, Pb, Hg, Cu, Cr, Cd, Zn, and As), reactive oxygen species (ROS), catalase activity (CAT), ultrastructural damage indexes (UDI, cytoplasm membrane, and chloroplast), and histopathological lesions (digestive tubules and ovotestis). Statistical analyses were performed using cage-level means (*n* = 3 cages per site).

Principal component analysis revealed clear multivariate patterns that descriptively separated exposure sites and bioindicator taxa. The first two principal components accounted for 96.0% of the total variance in the dataset, with PC1 and PC2 explaining 73.6% and 22.4%, respectively. Figure 10 (biplot) illustrates a distinct clustering pattern. The control samples (CTRL) formed a compact group independent of taxon, reflecting a shared baseline condition. In contrast, samples from the MM and GC sites were clearly separated from the controls and from each other along PC1. GC samples occupied the positive end of this axis, consistent with the higher levels of contamination documented at this site. Furthermore, moss and snail samples formed distinct subgroups within these site groups, suggesting different bioaccumulation dynamics and physiological responses to toxic elements. Variable load analysis showed that PC1 was strongly associated with key potentially toxic elements (Fe, As, Zn, Cr, and Cd) and biomarkers of oxidative stress (ROS and CAT). PC1 was also correlated with histopathological indices in snails (*I_h_* of the digestive tubules and *I_h_* of the ovotestis), supporting the interpretation that PC1 primarily reflects environmental pressure associated with metal accumulation and related biological stress responses, separating the control site from the two contaminated sites, while MM and GC differed along PC2. In contrast, PC2 was mainly defined by indices of ultrastructural damage in mosses (chloroplast UDI and cytoplasm/membrane UDI), with more modest contributions from Pb and Hg. In summary, PCA revealed three key findings: (*i*) sites were separated according to their contamination levels, with GC being the most impacted site; (*ii*) there were distinct, taxon-specific response profiles, indicating a potential complementarity between mosses and snails that requires further validation; and (*iii*) there was a consistent association between toxic elements accumulation, oxidative stress/histological damage in snails, and ultrastructural alterations in mosses.

## 3. Discussion

This study employed a combined bioindicator approach, utilizing the moss *R. squarrosus* and the land snail *C. aspersum*, to assess the levels of the biologically available fraction of atmospheric toxic elements at two sites in southern Italy with contrasting environmental characteristics. The MM site, which is located in a mountainous, inland area with low levels of urbanization and limited anthropogenic pressure, can be considered representative of a background exposure scenario. In contrast, the GC site is situated within the ‘Land of Fires’ area and is chronically affected by illegal waste disposal and recurrent fires, resulting in sustained atmospheric inputs of toxic elements. While environmental variables may influence exposure under fully natural conditions, these factors were controlled in the present experimental design. The moss and snails were exposed in suspended cages to avoid direct soil contact, and the snails were maintained under identical feeding and hydration conditions across the sites to minimize external variability and isolate site-specific atmospheric contamination signals. A key strength of this study is the combination of chemical, biochemical, histological, and spectroscopic endpoints across two taxonomic groups within a field-based framework [12,20]. This approach provides complementary information on different uptake pathways, which is particularly relevant in heterogeneous and chronically impacted environments, such as the ‘Land of Fires’ [11,21].

To quantify and integrate complex biological responses to exposure to toxic elements across taxa, a multivariate PCA analysis was performed (Figure 10). This approach explained 96% of the total variance in the first two components, providing a standardized comparison of samples and environmental conditions that supports the interpretation of tissue and cellular alterations associated with toxic element contamination. PC1 (73.6%) was strongly associated with metals such as Fe, As, Zn, Cr, and Cd, as well as with biomarkers of oxidative stress (ROS and CAT). This axis discriminated between the two environmental exposure scenarios by integrating both chemical contamination and associated biological responses [22,23]. The *I_h_* of the digestive tubules and ovotestis of snails also loaded strongly on PC1, indicating their sensitivity as target organs under exposure to toxic elements [24,25]. The *I_h_* scores showed a progressive trend, with more severe tissue alterations at the contaminated GC site. This is consistent with the digestive gland’s role as the primary organ for metal sequestration, detoxification, and biotransformation in mollusks, making it more susceptible to oxidative damage [26,27]. The alterations detected in the ovotestis suggest reproductive impairment with potential impacts on fertility, gametogenesis, and offspring viability [28,29,30]. PC2 (22.4%) captured more subtle sources of variation, primarily relating to ultrastructural damage in moss cells, such as chloroplast and membrane damage. This axis highlights the taxonomic complementarity of the system, with mosses enabling the early detection of subcellular damage following exposure to atmospheric toxic elements [31,32]. The ultrastructural damage index (UDI) confirmed the high sensitivity of *R. squarrosus* to alterations such as thylakoid disorganization, plastoglobule accumulation, electron-dense bodies, and plasmolysis. This further supports its suitability as an indicator of pollution pressure [11,33].

Morphometric analysis revealed that the albumen gland was significantly enlarged in snails from the GC site compared with those from the MM and control sites (Figure 8). This exocrine organ, which is essential for reproduction, secretes a galactose polymer that is used for egg capsule formation and embryo nourishment in snails [34]. While the relationship between metal exposure and albumen gland morphology remains unclear, prior research has indicated a potential correlation between gland enlargement and copper accumulation [35]. The enlargement observed in the present study is consistent with the higher copper concentrations measured in GC samples, which supports the hypothesis of metal-induced impairment of reproductive functions.

Biochemical analyses complementing these morphological changes showed a significant, dose-dependent increase in CAT activity in both mosses and snails, with the highest values recorded in GC samples. This is consistent with CAT’s role as an early and robust biomarker of metal-induced oxidative stress [27,36]. ROS levels exhibited a similar pattern, with a more pronounced increase between MM and GC in *C. aspersum* than in *R. squarrosus*, suggesting that snails are more responsive to increased metal loads. The absence of necrotic lesions in snail tissues, even under relatively high Cd exposure, may indicate the presence of compensatory physiological mechanisms, as has been observed previously in molluscs exposed to sublethal levels of metals [37,38].

In *C. aspersum*, both MM and GC environmental exposure induced comparable increases in HSP70 levels relative to controls, despite the substantially higher contamination at GC documented by other results. This pattern reflects a ceiling effect in the inducible stress response, where the MM contamination level was already sufficient to elicit maximal HSP70 induction, with no further upregulation possible under more severe exposure. Mechanistically, the heat shock response is governed by a negative feedback loop in which HSP70 represses its own transcription by regulating heat shock protein factors (Hsf1) activity [39]. Moreover, during chronic or repeated metal exposure, HSP70 expression has been shown to plateau after an initial peak, as demonstrated in terrestrial slugs exposed to heavy metals [40]. Thus, our 30-day exposure likely falls within a chronic timeframe where maximal induction already occurs at moderate contamination (MM). In contrast, CAT activity and histopathological damage accumulate progressively over time and do not show such homeostatic downregulation. HSP70 reflects the active cellular stress response at the time of sampling, whereas organ-level results integrate cumulative damage that does not diminish after 30 days. Therefore, the plateau in HSP70 does not indicate insensitivity of this molecular marker; rather, it suggests that the chaperone system is operating at full capacity. By contrast, *R. squarrosus* exhibited a progressive, dose-dependent increase in HSP70 levels. This species difference may reflect the less complex regulatory machinery in bryophytes, which lack the sophisticated feedback loops present in animals. Indeed, studies in aquatic mosses have shown that heavy metals induce a linear, concentration-dependent enhancement of HSP70 expression, supporting the use of HSP70 as a direct indicator of pollution load in plants [36].

FTIR spectroscopy applied to snail shells provided an additional dimension to complement the study. The spectral changes detected, including the reduction of the aragonite-associated bands and the appearance of new peaks in the fingerprint region, suggest possible alterations in shell mineralogy and/or organic matrix composition associated with environmental exposure to toxic elements. These additional peaks were absent in the spectra reported for non-contaminated snail shells by [41,42], which showed only the characteristic aragonite bands. However, additional elemental and mineralogical analyses would be required to confirm whether these spectral changes reflect direct metal incorporation into the aragonite matrix. In addition, the reduced intensity of aragonite bands may also reflect differences in calcification degree or changes in the aragonite/calcite ratio. Further analyses, such as X-ray diffraction (XRD), would help clarify these aspects. These findings highlight the potential of FTIR as a low-cost monitoring tool for detecting structural modifications in biomineral matrices associated with metal exposure [43,44]. Although the present study required destructive sampling, the analysis of empty shells collected in the environment could provide a non-lethal alternative for environmental monitoring.

Overall, the results suggest that a combined plant–animal bioindicator approach provides a more thorough characterization of environmental contamination and its impact on organisms. The studied organisms act as complementary bioindicators of atmospheric contamination, reflecting biological responses in organisms characterized by distinct contaminant uptake pathways as well as metal bioavailability and the associated physiological effects at the organ and cellular levels [45,46]. This represents a transferable framework for environmental assessment in contexts of widespread contamination [47,48]. Future studies could further expand this framework by: (*i*) extending exposure durations to capture long-term consequences for reproduction and development [12,49]; (*ii*) implementing seasonal sampling to incorporate temporal variability in pollutant dynamics [50]; (*iii*) increasing the number of study sites to develop a contamination gradient and including a point monitoring plan with passive air samplers or atmospheric dust collectors to obtain quantitative deposition measurement; and (*iv*) integrating targeted molecular biomarkers such as metallothioneins and phytochelatins, which would help elucidate detoxification pathways and mechanistic responses at the cellular level [51,52,53].

## 4. Materials and Methods

### 4.1. Experimental Setup and Samples

For the experimental setup, two study sites with different levels of environmental contamination were chosen: Montemiletto (41°01′00″ N, 14°54′00″ E) (MM) and Giugliano in Campania (40°55′34.4″ N, 14°05′58.5″ E) (GC). MM is located on the mountainous terrain that forms the watershed between the Calore and Sabato valleys in the central Irpinia hills, in the province of Avellino. This area is far from urban sources of pollution. In contrast, GC is situated in the north-western hinterland of Naples. It is one of the municipalities of the ‘Land of Fires’, an area between Naples and Caserta that has been affected by fires and illegal waste disposal. This has had serious consequences for the environment and public health [19]. In April 2024, approximately 300 g of moss were collected from the preserved natural oasis of Monte Nuovo (40°50′06″ N, 14°05′16.08″ E), located at an altitude of 133 m. Monte Nuovo is a nature reserve situated in the municipality of Pozzuoli and forms part of a protected area in the province of Naples. Once collected, the moss specimens were washed five times with distilled water to clean them of soil particles. The most viable samples were selected, divided into 1.5 g portions, and placed in 10 cm × 10 cm nylon nets with 1 mm^2^ mesh. These were then used for toxic elements evaluation. A pool of samples from this control site was used as the moss control sample.

Shortly before the experiment began, specimens of *Cornu aspersum* (formerly classified as *Helix aspersa*) were purchased from an environmentally preserved site in Santa Flavia, in the province of Palermo, Italy. Animals from this site were used as the control samples in this study. All specimens were sexually mature, weighing an average of 13.00 g, with an average shell diameter of 30 mm and height of 28 mm. Prior to placement in the study sites, the snails were sprayed with distilled water to emerge from their quiescent period and were fed ad libitum with lettuce (*Lactuca sativa*) and carrot (*Daucus carota*).

The specimens (mosses and snails) were exposed simultaneously under identical environmental conditions to ensure comparability of the experiment between the taxonomic groups. They were placed in drilled transparent plexiglass cages (20 × 40 × 60 cm) with 1 cm diameter holes to allow air to circulate during the exposure period. Three independent cages were placed at each site, 1.5 m above the ground. Each cage contained 24 snails and 8 moss bags. The cages were kept in situ for 30 days (1–31 May 2024), during which time the snails were water sprayed and fed four times a week. The 30-day exposure period was selected based on previous studies using the same biological models, which demonstrated that relevant physiological and biochemical responses could be detected within relatively short exposure windows [11,27]. Excreta and uneaten food were removed before hydration and feeding. A small container (10 × 8 cm) was placed inside each cage and filled with water to maintain constant humidity and prevent dormancy. The last feeding took place three days before sacrifice. The moss bags were sprayed at the same frequency as the snails to ensure adequate metabolic activity during the exposure period. At the end of the exposure period, the cages were removed from the sites. The surviving animals (24 per cage) were then processed for analysis: 18 snails from each cage (CTRL, MM, and GC) were stored at −80 °C for the analysis of trace elements in soft tissue and shells, as well as for biological analyses (quantification of reactive oxygen species (ROS) levels and antioxidant enzyme activity; HSP70), while six snails were destined for histological investigations. Similarly, six moss bags from each of the three sites were stored at −80 °C for trace element analysis and biological analyses, while two moss bags were destined for ultrastructural analysis by TEM (transmission electron microscopy, TECNAI G2, FEI INSTRUMENT, Hillsboro, OR, USA) (*n* = 3 cages per site).

### 4.2. Bioaccumulation

#### 4.2.1. ICP-OES Analysis

Samples of plant tissue from moss and soft tissue from land snails were collected. For each cage at each site, two moss bag samples and six snail soft tissue samples were analyzed. Data obtained from samples belonging to the same cage were subsequently averaged and used to calculate cage-level means. The samples were gently rinsed with deionized water to remove superficially adhered particles (without affecting internally bioaccumulated metals), then dried at 40 °C until constant weight to enable dry-weight normalization of metal concentrations. The dried materials were then homogenized using a non-metallic grinder to avoid contamination. For the metal analysis, approximately 0.3 g of each sample underwent acid digestion in a closed vessel using a high-purity acid mixture (HNO_3_ and HClO_4_) in a microwave digestion unit suitable for biological matrices (Milestone ETHOS UP). Each analytical batch included three procedural blanks, matrix-spike samples to verify recoveries, and certified reference materials (CRMs): IAEA-336 or BCR-482 for moss and NIST 2976 for animal tissue. Yttrium (Y) was added as an internal standard to all samples and calibration solutions to correct for instrumental drift. The digested solutions were analyzed using inductively coupled plasma optical emission spectrometry (ICP-OES, PerkinElmer Optima 8000, PerkinElmer, Inc., Waltham, MA, USA) in axial view mode to enhance sensitivity. Calibration curves were prepared using certified multielement standards, achieving coefficients of determination (R^2^) of at least 0.9995. The limits of detection (LOD) and quantification (LOQ), calculated according to the 3σ/10σ criterion, were consistent with the values commonly reported in the literature: Cd: 0.2–0.5 µg/L; Pb: 1–3 µg/L; Cr, Cu, Zn, Fe: 0.5–2 µg/L; As, Hg: 2–5 µg/L. Spike recoveries ranged from 88% to 107%, confirming analytical accuracy. All measurements were performed in triplicate, and the results were expressed on a dry-weight basis.

#### 4.2.2. FTIR Analysis

Snail shells are a biologically meaningful archive of metal exposure because trace elements can replace Ca^2+^ within the aragonite during shell formation. Therefore, FTIR-detected mineralogical changes reflect environmental contamination and organism-level physiological processes related to biomineralization and metal handling [40,41].

The shells removed from the snails used for bioconcentration analysis were analyzed using FTIR. They were washed and ground to produce fine shell powder (<125 μm), and KBr disks were made using 3.00 mg of the sieved sample and 197.00 mg of KBr. Analyses were also performed on control snail shells. FTIR analysis uses infrared light to scan test samples and obtain information about the nature of the chemical bonds present in materials. This technique enables functional groups within a molecule to be identified by analyzing their vibrational motions. FTIR analysis was performed using a Thermo Nicolet-6700 FTIR spectrometer (Thermo Fisher Scientific, Waltham, MA, USA) equipped with a deuterated triglycine sulfate (DTGS) detector in KBr. The resolution was 2 cm^−1^ (60 scans), with a spectral range of 400–4000 cm^−1^.

### 4.3. Biological Responses

#### 4.3.1. Protein Extraction and Quantification

Sample extraction for ROS determination and CAT activity assay and for Western blot analysis was performed using two distinct protocols. 

**Enzymatic assay extraction***:* for ROS and CAT 1 g from the greenest part of six moss bags from each cage were ground in liquid nitrogen and extracted with chilled Tris-HCl buffer (pH 7.5, 0.1 mM EDTA, 100 mM DTT), while snail hepatopancreas samples (12 for each cage) were extracted with chilled PBS buffer (pH 7.5, 0.1 mM EDTA). Then, samples were homogenized with tungsten microspheres using a TissueLyser and centrifuged at 6500 rpm for 30 min at 4 °C. 

**Western Blot extraction***:* for Western blot, snail samples were homogenized in RIPA buffer with protease inhibitors and centrifuged at 7000× *g* for 10 min at 4 °C, while moss samples were homogenized in RIPA buffer supplemented with sodium orthovanadate, PMSF, and protease inhibitors, then centrifuged at 14,000× *g* for 30 min at 4 °C. Protein concentrations were determined by Bradford PIERCE assay.

#### 4.3.2. ROS Quantification

The production of ROS was quantified by the oxidation of 2′,7′-dichlorofluorescein diacetate (DCFH-DA), according to protocols adapted from [18,54,55]. Extracts were incubated with a buffer solution containing DCFH-DA (40 µM) for 15 min at 37 °C. The esterases present in the supernatant hydrolyze DCFH-DA to DCFH, which is then oxidized by ROS to form the fluorescent compound DCF. The fluorescence intensity was measured with a microplate reader, using excitation/emission wavelengths of 485/535 nm. The results were expressed as intensity of fluorescence per milligram of fresh weight.

#### 4.3.3. Catalase Assay

CAT activity (units mg proteins^−1^) was kinetically measured (for 90 s at 25 °C, 30 s each read) as a decrease in the absorbance of H_2_O_2_ at 240 nm in 2 mL quartz cuvettes with a UV-Vis spectrophotometer. The reaction procedure was different in moss and snails. For mosses the reaction (1 mL reaction volume) started by adding 500 μL H_2_O_2_ (10 mM) to 40 μL of protein extract diluted in 460 μL Tris-HCl pH 7.5 buffer. For snails, the reaction (1 mL reaction volume) started by adding 500 μL H_2_O_2_ (10 mM) to 50 μL of diluted protein extract (1:5) in 450 μL PBS pH 7.8.

### 4.4. Western Blot for HSP70

For each sample extract, 80 μg of protein (40 μg to be loaded per gel) was denatured by boiling for 3 min in SDS buffer (50 mM Tris-HCl, pH 6.8, 2 g/100 mL SDS, 10% (*v*/*v*) glycerol, 0.1 g/100 mL bromophenol blue). The 12.5% SDS-PAGE gel was transferred to nitrocellulose membranes for quantitative analysis of the proteins using the Mini Trans-Blot system (Bio-Rad Laboratories). The membranes were kept at room temperature and subjected to a two-step blocking procedure, initially with 5% BSA in 1× TBS-T for 30 min. Rabbit polyclonal primary antibodies HSP70 (rabbit anti-Heat Shock Protein 70, Elabscience, Houston, TX, USA, E-AB-22005, 1:500), and GAPDH (rabbit anti-GAPDH, Elabscience, Houston, TX, USA, E-AB-40516, 1:2000) were used. Secondary antibody: goat anti-rabbit IgG (Sigma-Aldrich, St. Louis, MO, USA) was applied overnight at 4 °C at a 1:500 dilution in TBS-T containing 2.5% BSA. Protein bands were detected using a chemiluminescent HRP substrate (Amersham, Thermo Fisher Scientific, Milan, Italy) and visualized with the ChemiDoc Image System (Bio-Rad Laboratories, Hercules, CA, USA). Band intensities were quantified using ImageJ software (version 1.54g), and optical density (OD) values were normalized to GAPDH to account for loading differences.

### 4.5. Snails’ Histopathological Index (I_h_) and Albumen Gland Length Evaluation

Six snails were used for histological analysis of the digestive tubules and ovotestis from each cage at each study site. The samples were fixed by immersion in Davidson’s solution consisting of 11% glycerol, 22% formaldehyde solution (37%), 33.5% ethanol (95%) and 33.5% distilled water for 24 h at room temperature. The samples were washed daily in 70% ethanol to remove the fixative.

The snail samples were then dehydrated by passing them through a series of ethanol solutions of increasing concentration according to the following protocol: EtOH 50% for 15 min; two steps of EtOH 70% for 20 min; two steps of EtOH 85% for 20 min; two steps of EtOH 95% for 20 min; and two steps of EtOH 100% for 20 min. This was followed by one step of clarification in methyl benzoate for 48 h. The samples were then immersed in pure liquid paraffin in a vacuum oven at 58 °C. The paraffin was changed twice to remove any residual methyl benzoate. The samples were then embedded in clean paraffin and left to solidify overnight at room temperature. The samples were then cut into 10 µm thick sections using a rotary microtome and stained with Mayer’s hematoxylin [56].

The samples were observed using a light microscope (Leica DM RB, Wetzlar, Germany), and images were acquired from randomly selected fields of view using a digital camera (Canon PowerShot S50, Canon Inc., Tokyo, Japan), which was connected to the microscope and controlled via RemoteCapture software version 2.7.4.23. This was done to assess the degree of histological alterations (Table 3) [57,58,59]. To evaluate alterations induced by exposure to toxic elements present in the air, the histopathological index (*I_h_*) was calculated according to the formula proposed by [60]:

$$I_{h}=\frac{\sum_{1}^{j}w_{j}a_{jh}}{\sum_{1}^{j}M_{j}}$$

where *I_h_* is the histopathological index for h individuals; *w_j_* is the weight of the histopathological impairment; *a_jh_* is the value of the diffusion of alteration in the individual; and *M_j_* is the maximum diffusion of the alteration obtained by multiplying the weight by the maximum diffusion value. The denominator of the equation normalizes the indices to a value between 0 and 1, enabling comparisons to be made between different conditions. The weight (w) of each alteration ranges from 1 (minimum severity) to 3 (maximum severity), while the degree of diffusion varies from 0 (no alteration present) to 6 (widespread). To facilitate reproducibility, a step-by-step numerical example for the computation of $I_{h}$ is available in the Supplementary Materials. The indices were estimated for snail organs (digestive tubules and ovotestis). The alterations sought for the digestive tubules were an increase in excretory cells, atrophy, intertubular hemocyte infiltration, and intratubular cellular debris. For the ovotestis, the alterations sought were empty follicles, lipofuscin aggregates, disrupted architecture, and germ cell destruction [61]. The weight (w) of each alteration for the considered snail tissues is shown in Table 1. Finally, the enlargement of the albumen gland was measured with a caliper and expressed in cm.

### 4.6. Transmission Electron Microscopy: Moss Ultrastructural Damage Index (UDI)

Our TEM observations focused on leaflets from the subapical segments of two moss bags of gametophytes for each cage, cut out with a razor blade and located between 0.5 and 1.5 cm from the apexes. Five gametophytes were analyzed for each pouch, and ten observations were made for each sample. The samples were fixed in a 2.5% glutaraldehyde solution in a phosphate buffer solution (pH 7.2–7.4) overnight at 4 °C, then post-fixed with a solution of 1.5% osmium tetroxide and 0.8% potassium ferrocyanide for 1.5 h at room temperature. The samples were then dehydrated using an ethanol series and propylene oxide and embedded in Spurr’s epoxy resin. We cut 70 nm thick ultrathin sections and collected them on 200-mesh copper grids. Staining was performed using Uranyl Replacement Stain UAR (Electron Microscopy Sciences, Hatfield, PA, USA) and lead citrate. Micrographs were taken using a TECNAI G2 FEI instrument with an accelerating voltage of 120 kV and an Eagle 4K HR camera (FEI Company, Hillsboro, OR, USA). For each treatment, three independent TEM preparations were examined, each including multiple gametophytes.

To assess the alterations induced in mosses by exposure to toxic elements, we developed an ultrastructural damage index (UDI), based on the same principle as the histopathological index applied to snails in this study. The UDI quantified the cellular alterations observed by TEM in the gametophyte cells of *R. squarrosus* collected from both the experimental and the control sites. The following alterations were considered: disorganization of the thylakoid system and grana; presence and size of plastoglobules and starch granules in chloroplasts; plasmolysis; and presence of electron-dense bodies and multivesicular bodies in the cytoplasm (see Table 4). Each alteration was assigned a weight (w) from 1 to 3 according to its severity, while the extent of the alteration in the analyzed cells was assessed on a scale from 0 (absent) to 6 (maximum extent). The index was calculated according to the formula used for snails’ soft tissues, yielding a value between 0 and 1.

### 4.7. Statistical Analysis

Principal component analysis (PCA) was initially employed as a descriptive and exploratory tool to investigate the multivariate relationships between toxic elements and biochemical parameters across all experimental treatments (control, GC, and MM) and organisms (mosses and snails). The analysis was performed using the R statistical software package (version 4.2.3), utilizing FactoMineR for computation and FactoMineR Extra for visualization. To ensure that all variables contributed equally to the model, regardless of their measurement scales, the data were standardized to unit variance prior to analysis. The aim of the PCA was to reduce the dimensionality of the dataset, identify the key variables driving the observed multivariate structure, and visualize potential clustering of samples based on their overall profile. Cage-level means were used as the experimental unit (*n* = 3 cages per site). Measurements obtained from multiple moss bag and snail samples within the same cage were averaged prior to statistical analyses. The assumptions for parametric testing were then verified. The normality of all variables was confirmed using the Shapiro–Wilk test (*p* > 0.05), and the homogeneity of variances across groups was validated using the Brown–Forsythe test (*p* > 0.05). After confirming these assumptions, we assessed the effect of the different conditions using a one-way ANOVA, setting statistical significance at *p* < 0.05. Where the ANOVA indicated a significant effect, post hoc pairwise comparisons were conducted using the Tukey honest significant difference (HSD) test to control the family-wise error rate for multiple comparisons. The Pearson correlation between *R. squarrosus* and *C. aspersum* regarding single-element bioaccumulation was performed. All statistical analyses, excluding the preliminary PCA, were performed using GraphPad Prism (version 10.0, GraphPad Software, San Diego, CA, USA).

## 5. Conclusions

This study provides initial evidence supporting the feasibility of a combined plant–animal bioindicator approach involving the moss *R. squarrosus* and the land snail *C. aspersum*. Combining these species provided complementary information on biological effects associated with atmospheric toxic elements contamination. The high concordance in bioaccumulation patterns and increased oxidative stress (ROS and CAT) in snails, as well as ultrastructural damage in mosses, allowed the distinction between two contrasting environmental scenarios and identified the GC area as the most affected. Multivariate PCA synthesized these multifactorial responses descriptively, supporting the internal coherence of the integrated approach and the observed association between atmospheric exposure, bioaccumulation, and biological effects. While the results are consistent and promising, the correlative nature of this field study and its limited time scope highlight the need for more in-depth investigations, including mechanistic studies under controlled conditions, long-term seasonal monitoring, and integration of additional molecular biomarkers. Overall, this integrated approach may represent a promising complementary tool for more comprehensive, ecologically meaningful environmental assessments in areas subject to complex anthropogenic pressures.

## Figures and Tables

**Table 1:** Semi-quantitative classification of mean histopathological indices *I_h_* for all sites per target organ of snails according to the prevalence of histopathological alterations: 0–0.25, low; 0.25–0.50, moderate; 0.50–0.75, high; 0.75–1, very high. Different letters indicate significant differences among treatments according to Tukey’s HSD post hoc test. Statistical analyses were performed using cage-level means (*n* = 3 cages per site).

Target Organ	Site	*I_h_*	Classification
Digestive tubules	CTRL	0.20 ± 0.04 a	Low
	MM	0.36 ± 0.07 b	Moderate
	GC	0.77 ± 0.11 c	Very high
Ovotestis	CTRL	0.10 ± 0.02 a	Low
	MM	0.29 ± 0.02 b	Moderate
	GC	0.73 ± 0.04 c	High

**Table 2:** Semi-quantitative classification of mean Ultrastructural damage indices (UDI) for all sites per target cell compartments of moss according to the prevalence of ultrastructural alterations: 0–0.25, low; 0.25–0.50, moderate; 0.50–0.75, high; 0.75–1, very high. Different letters indicate significant differences among treatments according to Tukey’s HSD post hoc test. Statistical analyses were performed using cage-level means (*n* = 3 cages per site).

Target Cell Compartment	Site	UDI	Classification
Chloroplast	CTRL	0.15 ± 0.03 a	Low
	MM	0.38 ± 0.05 b	Moderate
	GC	0.82 ± 0.06 c	Very high
Cytoplasm/membrane	CTRL	0.18 ± 0.02 a	Low
	MM	0.41 ± 0.04 b	Moderate
	GC	0.85 ± 0.05 c	Very high

**Table 3:** Histopathological alteration observed in *Cornu aspersum* digestive tubules and ovotestis.

Organ	Histopathological Alteration	Weight (w)
Digestive tubules	Excretory cells increase	1
	Atrophy	2
	Intertubular hemocyte infiltration	1
	Intratubular cellular debris	1
Ovotestis	Empty follicles	1
	Lipofuscin aggregates	2
	Disrupted architecture	3
	Germ cell destruction	3

**Table 4:** Ultrastructural cellular alteration observed in moss *R. squarrosus*.

Cell Compartment	Cellular Alterations	Weight (w)
Chloroplast	Alteration of the thylakoid system and grana	2
	Presence and size of plastoglobules	1
	Presence and size of starch granules	1
Cytoplasm/membrane	Plasmolysis	2
	Presence of electron-dense bodies	3
	Presence of vesicles and multivesicular bodies	2

## Data Availability

The raw data supporting the conclusions of this article will be made available by the authors on request.

## Supplementary Materials

The following supporting information can be downloaded at: https://www.mdpi.com/article/10.3390/ijms27125225/s1.
