# Supplementary material for: Mosses and Snails as Bioindicators Reflecting the Biologically Relevant Fraction of Toxic Elements
Source: Int J Mol Sci. 2026 Jun 9;27(12):5225. doi: 10.3390/ijms27125225 (PMC13299311; doi:10.3390/ijms27125225)
Supplement: Supplementary file 1 [file ijms-27-05225-s001.zip › ijms-4309684-supplementary.pdf]

A

Cd g/kg					
**Samples**				**Average**	**Dev.St**
Ctrl	0,07	0,069	0,075	0,0713	0,003215
Montemiletto	0,11	0,1	0,099	0,103	0,006083
Giugliano	0,22	0,24	0,19	0,217	0,025166

Pb g/Kg					
**Samples**				**Average**	**Dev.St**
Ctrl	1,8	3	2,1	4,28	0,6245
Montemiletto	6,3	6,6	5,9	10,8	0,351188
Giugliano	15	15,2	16	15,4	0,52915

Fe g/kg					
**Samples**				**Average**	**Dev.St**
Ctrl	636	690	650	659	28,0238
Montemiletto	980	1013	999	997	16,56301
Giugliano	2800	2820	2700	2773	64,29101

Cr g/kg					
**Samples**				**Average**	**Dev.St**
Ctrl	2,57	2,46	2,61	2,55	0,077675
Montemiletto	3,12	3,56	3,13	3,27	0,251197
Giugliano	5,22	5,4	6	5,54	0,408412

As g/kg					
**Samples**				**Average**	**Dev.St**
Ctrl	0,7	0,9	1	0,867	0,152753
Montemiletto	1	1,03	0,98	1,00	0,025166
Giugliano	2,2	2,24	1,98	2,14	0,14

Cu g/kg					
**Samples**				**Average**	**Dev.St**
Ctrl	4.6	4,2	5,5	4,85	0,919239
Montemiletto	5	6,9	5,9	5,93	0,950438
Giugliano	14,22	16	15.6	15,1	1,25865

Zn g/kg					
**Samples**				**Average**	**Dev.St**
Ctrl	3	3.11	3,5	3,25	0,353553
Montemiletto	9,9	10,3	10,8	10,3	0,450925
Giugliano	90	92,4	99,8	94,1	5,108163

Hg g/kg					
**Samples**				**Average**	**Dev.St**
Ctrl	0,015	0.0178	0,013	0,0140	0,001414
Montemiletto	0,01	0,0103	0,03	0,0168	0,011461
Giugliano	0,058	0,078	0,06	0,0653	0,011015

B

Cd g/kg					
**Samples**				**Average**	**Dev.St**
Ctrl	0,099	0,1	0,087	0,0953	0,007234
Montemiletto	0,623	0,566	0,511	0,567	0,056003
Giugliano	1,13	1,01	1,17	1,103	0,083267

Pb g/Kg					
**Samples**				**Average**	**Dev.St**
Ctrl	2,98	3	2,76	5,047	0,133167
Montemiletto	7	7,98	6,56	15,1	0,726911
Giugliano	23,1	22	24	23,03	1,001665

Fe g/kg					
**Samples**				**Average**	**Dev.St**
Ctrl	712	719	777	736	35,67913
Montemiletto	1500	1456	1478	1478	22
Giugliano	3599	3578	3980	3719	226,2764

Cr g/kg					
**Samples**				**Average**	**Dev.St**
Ctrl	3,2	3,7	3,9	3,6	0,360555
Montemiletto	5	5,67	6,11	5,59	0,558957
Giugliano	10	11	11,7	10,9	0,8544

As g/kg					
**Samples**				**Average**	**Dev.St**
Ctrl	0,2	0,19	0,23	0,207	0,020817
Montemiletto	1	1,09	1,11	1,067	0,058595
Giugliano	4,5	4,199	4,78	4,49	0,290563

Cu g/kg					
**Samples**				**Average**	**Dev.St**
Ctrl	8	7,12	8,18	7,77	0,567215
Montemiletto	10	13	9,88	11,0	1,76771
Giugliano	35	37	35,122	35,7	1,121143

Zn g/kg					
**Samples**				**Average**	**Dev.St**
Ctrl	5	5,18	6,17	5,45	0,63
Montemiletto	30,5	28,2	35,11	31,3	3,518764
Giugliano	412	370	411	398	23,96525

Hg g/kg					
**Samples**				**Average**	**Dev.St**
Ctrl	0,007	0,008	0,0012	0,01	0,003672
Montemiletto	0,02	0,012	0,016	0,02	0,004
Giugliano	0,082	0,089	0,079	0,08	0,005132

Figure S1. Bioaccumulation values (g/kg) of the elements measured in *C. aspersum* (A) and *R. squarrosus* (B) after 30 days of exposure.

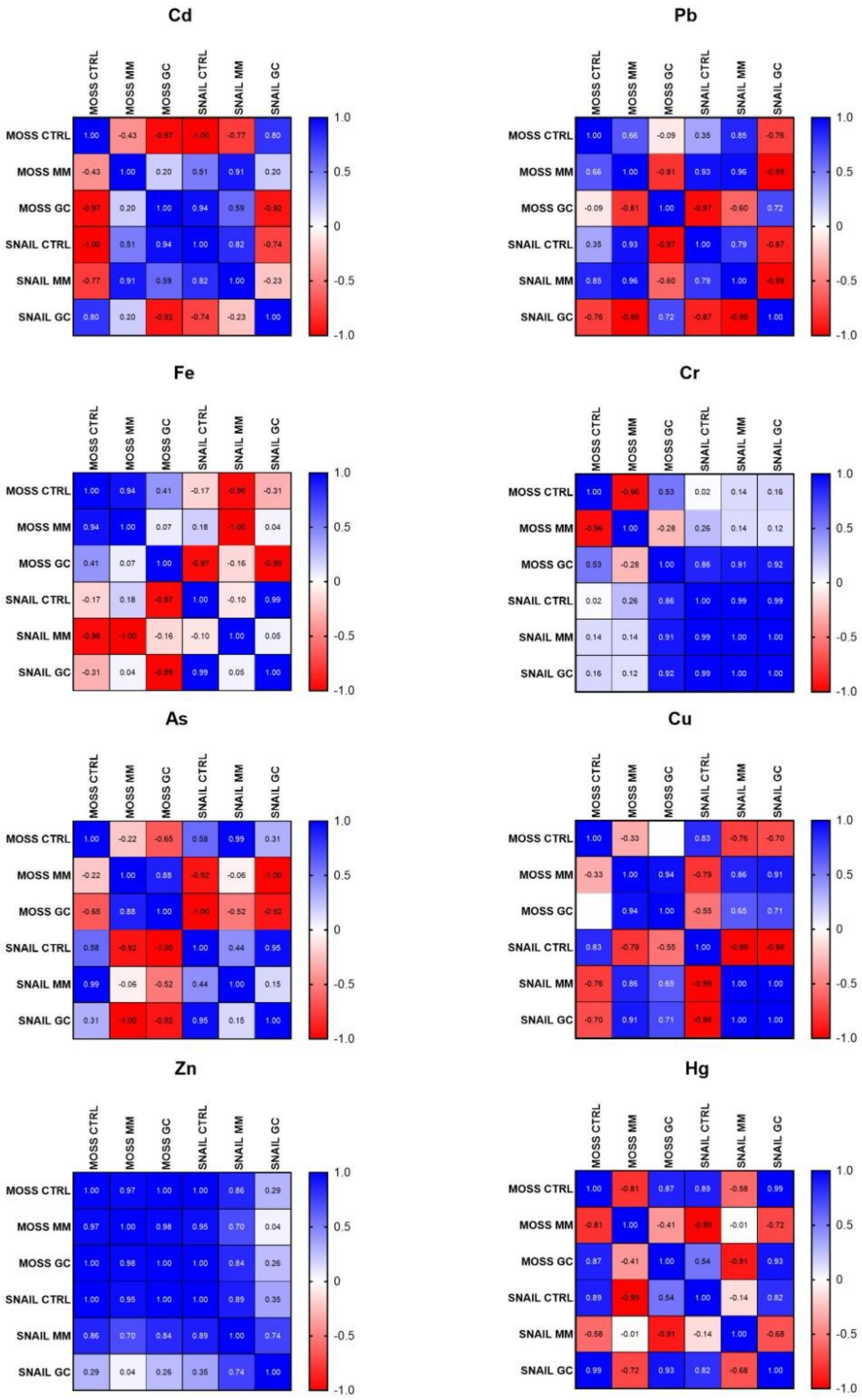

Figure S2. Pearson correlation heatmaps for the concentrations of heavy metals (Cd, Pb, Fe, Cr, As, Cu, Zn, and Hg) in moss *R.squarrosus* and snail *C. aspersum* samples under different environmental conditions: control (CTRL), Monte Miletto (MM), and Giugliano in Campania (GC). Correlation coefficients (r) range from −1 to +1 and are represented by a color scale: blue indicates positive correlations, red indicates negative correlations, and white indicates no correlation. Values close to +1 indicate a strong positive association among treatments, whereas values close to −1 indicate a strong inverse relationship.

Detailed Worked Example: Calculation of the I_h Index: For a snail from the GC site, digestive tubule alterations might include: excretory cells increase (w=1, diffusion=4), atrophy (w=2, diffusion=3), hemocyte infiltration (w=1, diffusion=5), and cellular debris (w=1, diffusion=2). Numerator = (1×4)+(2×3)+(1×5)+(1×2)=17. Denominator = (1×6)+(2×6)+(1×6)+(1×6)=30. I_h = 17/30 ≈ 0.57 → "high" damage (Table 1).
